# How Do the Chinese Perceive Ecological Risk in Freshwater Lakes?

**DOI:** 10.1371/journal.pone.0062486

**Published:** 2013-05-09

**Authors:** Lei Huang, Yuting Han, Ying Zhou, Heinz Gutscher, Jun Bi

**Affiliations:** 1 State Key Laboratory of Pollution Control & Resource Reuse, School of the Environment, Nanjing University, Nanjing, P.R. China; 2 Department of Environmental Health, Rollins School of Public Health, Emory University, Atlanta, Georgia, United States of America; 3 Department of Psychology, University of Zurich, Zurich, Switzerland; University of Sydney, Australia

## Abstract

In this study, we explore the potential contributions of a risk perception framework in understanding public perceptions of unstable ecosystems. In doing so, we characterize one type of common ecological risk– harmful algal blooms (HABs)–in four of the most seriously eutrophicated freshwater lakes in China. These lakes include Chaohu, Dianchi, Hongze, and Taihu, where a total of 2000 residents living near these sites were interviewed. Regional discrepancies existed in the pilot study regarding public perceptions of ecological changes and public concerns for ecological risk. Comparing HABs and other kinds of risks (earthquake, nuclear, and public traffic) through the psychometric paradigm method, *Knowledge*, *Effect*, and *Trust* were three key factors formulating the risk perception model. The results indicated that *Knowledge* and risk tolerance levels had significant negative correlations in the higher economic situation while correlations in the lower economic situation were significantly positive. *Effect* and risk tolerance levels had significant negative correlations in the high and middle education situation while correlations in the low education situation were close to zero or insignificant. For residents from Taihu with comparatively higher economic and educational levels, more investment in risk prevention measures and stronger policies are needed. And for residents from Hongze and Dianchi with comparatively low economic and educational levels, improvement of the government’s credibility (*Trust*) was the most important factor of risk tolerance, so efforts to eliminate ecological problems with the stepwise development of economic and educational levels should be implemented and gradually strengthened. In turn, this could prevent public discontent and ensure support for ecological protection policies.

## Introduction

As a result of anthropogenic activities such as aquaculture, agriculture, waste discharges from industry, and human recreational activities [1], the rising ecological risk of harmful algal blooms (HABs) which may cause human disease [2] has become a worldwide concern [3], especially regarding China. HABs currently disturb four of China’s largest fresh water lakes: Chaohu, Dianchi, Hongze, and Taihu, leading to different levels of water quality degradation, ecological damage, threats to human health, and socioeconomic losses [4], [5]. An algal bloom event which contaminated 70% of the water plants in Wuxi attracted extensive national attention in June 2007; drinking water contamination prevented nearby 2 million citizens from obtaining potable water for several days, which resulted in excess demand for bottled water and consequently price inflation (nearly fivefold) [3]. Citizens may have varying levels of sensitivity to the ecological risks posed by frequent HABs, and there exists a diverse array of public risk perceptions regarding this kind of risk. Thus, risk perception research helps us to ascertain the attitudes of the public regarding ecological risk and to understand the factors that determine risk tolerance. Depending on career [6], [7], education [8], [9], gender [10], [11], *etc.,* individuals hold different perceptions on how dangerous an ecological risk could be. In addition to scientific and technical information [12], other factors (experience, familiarity, residency, etc.) influence individual decision-making abilities [2], [6], [13]–[15]. The psychometric paradigm method was first used in a risk analysis of a nuclear power plant [8], and it is currently the most influential method that sociologists apply in the field of risk analysis of public perception [16]. The “cognitive map” of hazards produced by the paradigm explains how people perceive the various risks they face [17], which uses several hazard characteristics (e.g., controllability, newness, dreadfulness, etc.) that hypothetically influence risk perception. Previous risk analysis studies [18] have surveyed a wide range of hazardous events which were divided into 4 main types [19]: technical (e.g., nuclear power plants), ecological (e.g., global warming), daily (e.g., public traffic), and natural (e.g., floods, landslides, earthquakes) hazards. These studies revealed the various factors that can significantly influence risk perception, such as how one’s familiarity with public transportation impacts the degree of risk tolerance more significantly than one’s familiarity with nuclear power. Furthermore, risk perception was influenced by individual-difference predictor variables, including demographics [10], [11], [20]–[23], expertise [7], [8], [24], the potential ecological impact [25]; social background (i.e. culture) [26], customs [27], economic development status [14], and environmental conditions [28], [29], all of which could also affect public actions and perceptions [30]–[32].

Currently, few studies have discussed the impact of lake ecological risks on public perception, and none have taken into account regional discrepancies. Regarding the public attitudes towards water quality, some considered clear water as the most important water quality characteristics followed by fewer HABs [33] and were willing to pay for a reduction in the health risks posed by HABs [34]. However, there are still some people who fail to consider the chronic effects, which result in their perceived risk different from the actual [35], [36]. As public risk perception is likely to strongly influence behavior and result in different risk levels [35], improving risk perception through education for risk management and reduction seems very important. China has numerous lakes which are also vital drinking water resources, and the distribution of these lakes is widely dispersed. Understanding public risk perception can greatly inform policy officials on what constitutes publicly-acceptable water management strategies, while maintaining the ecological health of vital water bodies. Due to the 2007 algal-bloom outbreak in the Lake Taihu region, on September 7, 2011 the State Council promulgated the Regulation on the Administration of Taihu Lake Basin. In this way, the actions of the State Council underscored the dramatic importance of balancing ecological protection policies with local political governance [37]. Thus it is worth studying how the Chinese perceive ecological risks in these lake regions.

The present work aims to explore the potential contributions of a risk perception framework in understanding Chinese public perceptions of unstable ecosystems. In doing so, we use HABs as a primary example from which to characterize a lake ecological risk. This study aims to discover the following: 1) determining factors which influence public tolerance in the risk perception model by using a comparative analysis of four typical kinds of hazards; 2) perception factors which create discrepancies in local residents’ risk tolerance levels of HABs; 3) and how demographic characteristics and social indicators influence the relationship between each perception factor and risk tolerance level.

## Methods

### Ethics Statement

This study was approved by the review board of Nanjing University. All participants were informed about the objectives and methods of the study before the investigation and written consent was obtained from all participants. The study did not involve participants from abroad.

### Samples and Data Collection

**Figure 1:**
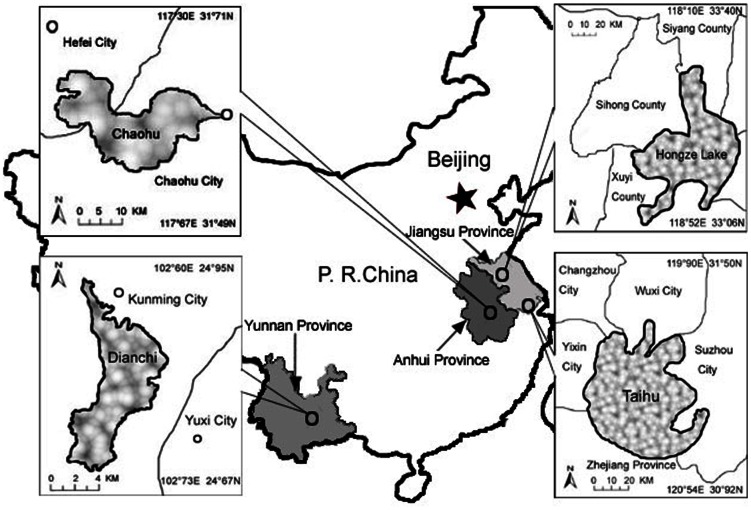
Location of investigating area and four large lakes in China.

In this study, we focused on the ecological risks posed by HABs that could cause human disease at four of the largest and most eutrophicated lakes in China, which include Chaohu, Dianchi, Hongze, and Taihu. The respondents were selected by a stratified random sampling of those living in cities or counties around these lakes. Each city or county was divided into several districts, with each district further divided into residential communities. Households within these randomly selected communities were randomly selected. Fig. 1 shows the locations of these four sampled areas.

Questionnaire designs and surveys were conducted at all of the four lake areas in 2008. A total of 2,000 adults were surveyed based on sample proportions from large populations [38], as well as 120 adults in pilot study, and each area included 500 respondents. Because some respondents did not complete the survey or skipped questions, only 1,361 of the sampled respondents were qualified to be used in our analysis. The samples matched the respective populations well in terms of demographics, with ‘education’ as the exception. High level education (>12 years) was overrepresented in the survey compared to local demographics, which was coincident with many previous studies [14], [29], [39]. This phenomenon can be attributed to the fact that people with higher levels of education are more likely to participate in survey studies and complete questionnaires [40]. There is a similar overrepresentation of high education levels in each lake investigation; thus, the bias of the samples was likely of minor impact on the results of the comparison analysis among the four lakes. The survey for Lake Chaohu was conducted from July 2 to July 8, 2008, and 339 questionnaires qualified for analysis (a response rate of 68%). The survey for Lake Dianchi was conducted from July 10 to July 17, and 267 qualified (a response rate of 53%). The survey for Lake Hongze was conducted from July 18 to July 25, and 315 completed questionnaires were filled out (a response rate of 62%). The survey for Lake Taihu was conducted from August 1 to August 9, creating a total of 440 completed questionnaires (a response rate of 88%). Please see Table S1 for detailed results of the surveys. The surveys were conducted at the respondent’s workplace or home, and about 30 minutes were needed to finish all questions. Twenty senior students from Nanjing University’s School of Environment conducted the survey. Before the start of the survey, they were trained in research methods and survey techniques. If the respondents were unclear about the questions, the students conducting the surveys could provide explanations but were forbidden from giving answers to the questions.

The survey collected information on four aspects: perceptions of ecological change, perceptions of risk characteristics, demographic variables, and social indicators. The first three were investigated by questionnaire survey. In this study, the questionnaire design was adapted from Slovic et al. [8], focusing on HABs and for reasons of comparison, three other kinds of hazards with typical risk characteristics: earthquakes (natural disaster), nuclear power (technical and fatal hazards), and public traffic (daily and familiar hazards). In addition, referring to the research of Sjöberg [29], possible risk characteristic variables that could be included in risk perception models were also selected (i.e., newness, immediacy, social risk, personal impact, knowledge, benefit, dread, and trust in government). On the basis of the pilot study, the questionnaire structure is proven valid and identical to Bartlett’s Test of Sphericity. Then we modified vague expressions and created the formal questionnaire (Questionnaire S1). Perceptions of ecological change and risk characteristics were evaluated on a five-point Likert scale [8], [11], [16], [29]. The demographic variables included sex, age, education, occupation, and annual income. Social indicators included socio-economic status, socio-educational level, and socio-environmental condition, most of which were derived from official statistics and fieldwork results [38], [41], [42]. Social indicators selected by extensive comparative and screening analysis with literature references [8], [22], [23], [29], [30], [43], [44] were used to analyze associations within tolerable risk levels and risk perceptions. The socio-economic status of each city or county in the researched lake areas could be expressed in three ways: GDP per capita, energy consumption per capita, and proportion of urban population. There were two variables in the socio-educational level: proportion of advanced education degrees and the quantity of media consumption (i.e., TV, radio, newspaper, etc.). Socio-environment conditions included the degree of lake service function changes (i.e., domestic water, industrial water, agricultural water, etc.) and a number of high-polluting enterprises. The data of variables depicted above come from local statistical yearbooks [38], [41], [42] except proportion of lake service function changes which is based on data from Lake (Chaohu, Dianchi, Hongze, and Taihu) Ecosystem Services Assessment Report (Table S2).

### Data Analysis

Two statistical analyses were performed in this study. First, we analyzed the determining factors of ecological risk perception, comparing it to the three different kinds of hazards mentioned above (HABs compared to earthquakes, nuclear power, and public traffic). Comparisons between risk characteristics (*Risk tolerance, Newness, Immediacy, Social risk, Personal effect, Knowledge, Benefit, Dread, Trust*) of four kinds of hazards and four lakes were tested by the one-way ANOVA method. Following the traditional psychometric approach, a maximum likelihood factor analysis with oblique promax rotation was used to find the risk perception factors associated with each hazard. Three steps helped to identify the differences in perception of HABs in the four different lakes: factor analysis was conducted to find the risk perception factors, followed by a correlation analysis to test the validity of the factor analysis. The third step involved a multiple regression analysis to examine the determining factors of risk perception.

Second, a Kruskal-Wallis H test was conducted to examine regional demographic diversities of the researched areas, and the relationships between selected social indicators and risk tolerance were analyzed. In addition, factor space analysis [27], categorized by groups of people, was used to determine how demographic characteristics or social indicators influence the relationship between risk tolerance levels of HABs and each determining risk perception factor. We used these correlations to determine unit-length risk tolerance vectors for the participants in the given-samples factor space. The open symbols are projections of the endpoints of these vectors onto the three planes defined by pairs of oblique factors. Long projections (endpoints far from the origin) indicate that risk tolerance levels of HABs are highly correlated with the factors that define the plane, whereas short projections (endpoints close the origin) indicate that risk tolerance levels are not highly correlated with the same factors. The cosine of the angle between a pair of factors is equal to the correlation between those factors. Positive and negative correlations between vectors and factors can be judged relative to the dashed lines perpendicular to the factors. In this study, the length of the factor axes from the origin is 1.0. The symbols in different sizes and colors represent the risk tolerance level vectors for different groups of respondents for each demographic characteristic or social condition. The data analysis and reliability testing were performed by statistical package SPSS 17.0.

## Results

### Pilot Study on Public Perception of Ecological Changes

Table 1 provides background information on the changes that the four lakes have undergone in recent decades, showing deteriorating water quality and ecological changes occurring in each lake. There are both similarities and differences in perceived ecological changes in recent decades among residents living near the four lakes (see Table 2). The most noticeable phenomenon is that the residents from all lakes felt HABs occurred much more frequently, especially residents from Lake Taihu, who nearly unanimously agreed that HABs occur more frequently than the previous ten years (M = 4.12, SD = 1.17). As shown in Table 1, the water quality of Dianchi was the worst of all the lakes we investigated; however, Chaohu, Hongze and Taihu have greater deteriorated changes than Dianchi in the recent 10 years. In addition, some irrigation works brought great changes to Lake Hongze (e.g., lake volume shrank by a third) [44], [45], but the residents did not perceive as strong a risk as those from Lake Taihu, while the actual changes of Lake Hongze was bigger than Lake Taihu in recent 10 years. Therefore, we must ascertain the key factors that impact public sensitivity and cause differences in individual perceptions of ecological changes.

**Table 1:** The Background of Four Lakes [44], [45], [58].

Year	Chaohu	Dianchi	Hongze	Taihu
1983	22%II;78%III	100%V	18%II;50%III;32%IV	69%II;19%III;12%IV
1993	6.5%III;13.6%IV;79.9%V	100% worse than V	33.3%III;66.7%IV	16.3%II;75.5%III; 8.2%IV
2003	50%V;50%worse than V	100% worse than V	100%V	14.3%IV;14.3%V; 71.4%worse than V
Area	769 km^2^	330 km^2^	2069 km^2^	3100 km^2^
Surrounding citiesand counties	Hefei, Chaohu	Kunming, Yuxi	Sihong, Xuyi, Siyang	Wuxi, Suzhou, Changzhou, Yixing

Note: Descriptions of II, III, IV, and V are shown in Table S3.

**Table 2:** Public Perception of Ecological Change of Four Lakes.

Survey questions	Chaohu(N = 339)		Dianchi(N = 267)		Hongze(N = 315)		Taihu(N = 440)	
	Average	SD	Average	SD	Average	SD	Average	SD
Water quality^a^	3.80	1.45	3.57	1.52	3.21	1.60	3.96	1.45
HABs^b^	3.81	1.44	3.53	1.48	3.15	1.44	4.12	1.17
Area^c^	3.57	1.25	3.61	1.32	2.97	1.38	3.40	1.40
Fish^d^	3.92	1.41	3.92	1.39	3.57	1.65	4.02	1.23
Birds^e^	3.87	1.22	3.55	1.41	3.93	1.06	4.09	1.08
Water plants^f^	3.66	1.36	3.56	1.44	3.17	1.57	3.46	1.51
Tourists^g^	2.37	0.97	1.79	0.81	1.93	0.85	1.95	1.02
Inhabitants^h^	2.14	1.31	2.28	1.01	3.05	1.42	1.77	0.71

a “Do you feel that the water quality of the lake has changed in the last decade?” Scale ranges from “much improved” (1) to “much worsened” (5);

b “Have you noticed that the lake’s harmful algal blooms are occurring more frequently than a decade of ago?” Scale ranges from “strongly disagree” (1) to “strongly agree” (5);

c “Do you feel that the area of wetlands surrounding the lake has changed in the last decade?” Scale ranges from “much increased” (1) to “much decreased” (5);

d “Do you feel that the number species of fish in the lake has been changed in the last decade?” Scale ranges from “much increased” (1) to “much decreased” (5);

e “Do you feel that the number of species of birds inhabiting this lake has changed in the last decade?” Scale ranges from “much increased” (1) to “much decreased” (5);

f “Do you feel that the number of species of water plants in the lake has changed in the last decade?” Scale ranges from “much increased” (1) to “much decreased” (5);

g “Do you feel that the number of tourists to this lake has changed in the last decade?” Scale ranges from “much increased” (1) to “much decreased” (5);

h “Do you feel that the number of inhabitants living around the lake has changed in the last decade?” Scale ranges from “much increased” (1) to “much decreased” (5).

### What are the Determining Factors that Influence Public Tolerance in the Risk Perception Model?

#### Comparison between HABs and three other hazards

Earthquakes are natural disasters that have immediate impact. Nuclear power is associated with involuntary, unknown, new, uncontrollable, and fatal risk characteristics. The risk of public traffic is a daily hazard with which people are familiar. Compared with these risks, HABs are hazardous ecological events that could afflict water bodies (eutrophic lake, coastal ecosystems, etc.) on an almost global scale and exert a long-lasting effect on the environment. The tolerable risk level of earthquakes was ranked last in the comparison analysis of risk tolerance, the frequency of which usually is regarded as a risk background value. However, such a low tolerable level of the respondents in this survey was thought mainly due to the Wenchuan Earthquake in China that occurred just two months prior to the date of the survey (May 12^th^, 2008). Table 3 shows that the four hazards had significantly diverse characteristics including *newness* (F = 27.30, p<0.001), *immediacy* (F = 12.52, p<0.001), *social risk* (F = 15.21, p<0.001), *personal effect* (F = 17.64, p<0.001), *knowledge* (F = 19.38, p<0.001), *benefit* (F = 11.79, p<0.001), and *dread* (F = 5.45, p = 0.014). The results of a rotated factor pattern are shown in Table 4. All attributes related to *newness* and *knowledge* were loaded on Factor 1 (labeled as *Knowledge*). *Immediacy*, *social risk, personal effect,* and *dread* were loaded on Factor 2 (labeled as *Effect*). Attributes related to *benefit* and *trust* were loaded heavily on Factor 3 (labeled as *Trust*). However, there were still a few minor differences among the hazards in the results of the factor analysis. For example, the characteristic variable *dread* of the earthquake hazard was split between Factor 2 and Factor 3, which means that the degree of *dread* of earthquakes was not only influenced by *Effect* but also by *Trust* in the government or organizations. The variable *social risk* on public traffic was also split between Factor 2 and Factor 3, revealing that public traffic was not only influenced by *Effec*t but also by *Trust* in the government or organizations. The variable of *dread* in the hazard of nuclear power was split between Factor 1 and Factor 2, which implies that the degree of *dread* the public holds for nuclear power plant is related to their *knowledge* about the plant; however, factor analysis revealed that *Effect* is still the primary factor.

**Table 3:** Descriptive Statistics of the Risk Characteristic Variables of Four Kinds of Hazards.

Variables	HABs	Earthquake	Nuclear Power	Public Traffic	One-Way ANOVA(F value)
	Men(SD)	Mean(SD)	Mean(SD)	Mean(SD)	
Risk tolerance^a^	2.28(1.22)	1.94(1.37)	2.03(1.31)	2.85(1.39)	21.49***
Risk characteristic variables affecting risk perception					
1. Newness^b^	2.38(1.48)	4.12(1.39)	2.51(1.46)	2.24(1.37)	27.30***
2. Immediacy^c^	3.03(1.64)	2.78(1.67)	2.09(1.60)	3.15(1.55)	12.52***
3. Social risk^d^	2.99(1.49)	2.47(1.49)	3.61(1.37)	2.16(1.44)	15.21***
4. Personal effect^e^	3.10(1.05)	2.56(1.21)	3.38(1.19)	2.84(1.37)	17.64***
5. Knowledge^f^	2.76(1.41)	3.03(1.51)	2.22(1.33)	3.49(1.40)	19.38***
6. Benefit^g^	1.60(1.11)	1.64(1.21)	2.19(1.41)	3.02(1.56)	11.79***
7. Dread^h^	3.07(1.53)	3.19(1.73)	1.63(1.50)	3.20(1.45)	5.45*
8. Trust^i^	2.34(1.36)	2.80(1.55)	2.12(1.46)	2.72(1.37)	1.43

a “If your life or working surroundings contain this kind of risk, what is your tolerable degree of risk?” Scale ranges from “Not tolerable at all” (1) to “Very tolerable” (5);

b “Is the risk associated with each activity, substance, or technology new and non-familiar, or is it old and familiar? ” Scale ranges from “Old” (1) to “New” (5);

c “Are the effects of the risk associated with each activity, substance, or technology immediate, or will they take place in the future?” Scale ranges from “Occurs immediately” (1) to “Occurs far in the future” (5);

d “How much risk is the national population subjected to as a product of each activity, substance, or technology?” Scale ranges from “No risk” (1) to “High risk” (5);

e “In what degree are you personally affected by the risk associated to each activity, substance, or technology?” Scale ranges from “Doesn’t affect me” (1) to “Affects me” (5);

f “To what degree is the risk associated with each activity, substance, or technology known to you?” Scale ranges from “No knowledge” (1) to “High level of knowledge” (5);

g “How beneficial to you is the use, consumption, or accomplishment of each activity, substance or technology?” Scale ranges from “Low” (1) to “High” (5);

h “Is the risk associated with each activity, substance, or technology a common risk or a terrible risk?” Scale ranges from “Common” (1) to “Terrible” (5);

i “To what degree do you trust in the government or organizations?” Scale ranges from “No Trust at all” (1) to “Complete trust” (5).

* p<0.05;

*** p<0.001.

**Table 4:** Factor Analysis Results of Risk Characteristics about Four Typical Kinds of Risks.

Variables	HABs			Earthquake			
	Total VarianceExplained = 78%			Total VarianceExplained = 76%			
	Factor1*Knowledge*	Factor2*Effect*	Factor3*Trust*	Factor1*Knowledge*	Factor2*Effect*	Factor3*Trust*	
Newness	**0.720**	−0.158	0.147	**0.661**	0.254	0.208	
Immediacy	0.079	**0.648**	−0.066	−0.139	**0.615**	0.226	
Social risk	−0.010	**0.764**	0.007	0.046	**0.726**	0.312	
Personal effect	0.052	**0.797**	0.201	−0.023	**0.812**	0.160	
Knowledge	**0.643**	−0.101	−0.008	**0.579**	0.325	−0.008	
Benefit	0.051	0.013	**0.619**	0.207	0.353	**0.768**	
Dread	0.200	**0.672**	0.251	−0.080	**0.489**	***0.411***	
Trust	−0.142	−0.067	**0.693**	***0.403***	0.121	**0.616**	
**Variables**	**Nuclear power**			**Public Traffic**			
	**Total Variance** **Explained = 75%**			**Total Variance** **Explained = 81%**			
	**Factor1** ***Knowledge***	**Factor2** ***Effect***	**Factor3** ***Trust***	**Factor1** ***Knowledge***	**Factor2** ***Effect***	**Factor3** ***Trust***	
Newness	**0.527**	0.341	0.082	**0.784**	0.208	−0.075	
Immediacy	0.089	**0.603**	0.154	0.170	**0.637**	−0.037	
Social risk	0.095	**0.653**	0.184	0.029	**0.793**	***0.439***	
Personal effect	0.105	**0.722**	0.028	0.009	**0.805**	0.037	
Knowledge	**0.643**	−0.077	0.217	**0.681**	−0.059	0.147	
Benefit	0.125	0.146	**0.643**	−0.078	0.257	**0.628**	
Dread	***0.413***	**0.474**	0.022	0.303	**0.635**	0.207	
Trust	0.109	−0.139	**0.625**	0.048	0.059	**0.757**	

Note: See Table 3 for variable description. |Factor pattern|>0.40 is in boldface type.

Although the types of hazards were significantly different from each other, we found that a three-factor solution (*Knowledge*, *Effect* and *Trust*) explained 75%–81% of the total variance in each hazard’s characteristics. Therefore, the selected risk factors appropriately described the hazards, but their impacts on public perception and risk tolerance of each hazard were diverse.

#### Correlation analysis of characteristic variables for HABs

The first item in Table 5 shows the respondents’ tolerance levels of HABs, implying that the residents around Lake Hongze and Lake Chaohu were comparatively willing to accept HABs risk (M = 2.58, SD = 1.24; M = 2.36, SD = 1.20, respectively), while the residents around Lake Taihu were the most reluctant to accept the risk (M = 1.97, SD = 1.19). It should be noted that risk tolerance levels of HABs were significantly different among the four lakes (F = 19.07, p<0.001), as were all the risk characteristic variables of HABs, except benefit (F = 2.09, p = 0.182). Differences on a pairwise basis between four lakes can be found in Table 6.

**Table 5:** Descriptive Statistics of the Risk Characteristic Variables of Four Large Lakes in China.

Variables	Chaohu(N = 339)	Dianchi(N = 267)	Hongze(N = 315)	Taihu(N = 440)	One-Way ANOVA(F value)
	Mean(SD)	Mean(SD)	Mean(SD)	Mean(SD)	
Risk tolerance	2.36(1.20)	2.09(1.26)	2.58(1.24)	1.97(1.19)	19.07***
Risk characteristic variables affecting risk perception					
1. Newness	3.74(1.54)	3.85(1.42)	3.10(1.53)	3.74(1.38)	18.99***
2. Immediacy	3.46(1.64)	2.93(1.68)	2.57(1.56)	3.16(1.60)	16.73***
3. Social risk	3.24(1.53)	3.00(1.54)	2.54(1.46)	3.17(1.40)	14.59***
4. Personal effect	3.12(1.27)	2.89(1.45)	2.70(1.32)	3.58(1.66)	19.42***
5. Knowledge	2.65(1.32)	2.65(1.41)	2.32(1.30)	3.21(1.41)	28.53***
6. Benefit	1.48(1.01)	1.69(1.21)	1.64(1.10)	1.58(1.10)	2.09
7. Dread	2.99(1.54)	2.92(1.50)	2.75(1.48)	3.46(1.51)	15.99***
8. Trust	2.13(1.29)	2.43(1.36)	2.50(1.40)	2.29(1.38)	4.34**

Note: See Table 3 for variable description.

** *p*<0.01;

*** *p*<0.001.

**Table 6:** The Differences of Risk Characteristic Variables on a pairwise basis between Four Lakes.

Variables	Chaohu			Dianchi			Hongze	Taihu
	& Dianchi	& Hongze	& Taihu	& Hongze		& Taihu	& Taihu	–
Risk tolerance	–0.27**	0.15	–0.36***	–0.52***		–0.13*	0.61***	–
Risk characteristic variables affecting risk perception								
1. Newness	–0.11	–0.75***	–0.11		–0.64***	0.004	0.64***	–
2. Immediacy	–0.53***	0.36*	–0.23		0.89***	0.30	–0.59***	–
3. Social risk	–0.24	0.46***	–0.17		0.69***	0.07	–0.63***	–
4. Personal effect	–0.23	0.65*	0.49**		0.26	–0.82***	–0.88***	–
5. Knowledge	0.002	0.33*	–0.56***		0.33*	–0.56***	–0.89***	–
6. Benefit	0.22	0.05	0.11		–0.17	0.11	0.06	–
7. Dread	–0.06	0.17	–0.53***		0.23	–0.47***	–0.71***	–
8. Trust	0.29*	–0.08	0.14		–0.37**	–0.16	0.22	–

Note: See Table 3 for variable description. *p<0.05;

** *p*<0.01;

*** *p*<0.001.

Many risk characteristic variables were correlated with each other [6], [15], [18], [19]. For example, *knowledge* correlated with both *social risk* (a = 0.190, p<0.01) and *newness* (a = −0.315, p<0.01), and *social risk* correlated with *immediacy* (a = 0.336, p<0.01). In particular, *dread* correlated with most variables, including *newness* (a = 0.234, p<0.01), *immediacy* (a = 0.240, p<0.01), *social risk* (a = 0. 367, p<0.001), *personal effect* (a = 0.322, p<0.001), and *benefit* (a = −0.144, p<0.05), so factor analysis was necessary to eliminate inter-correlation.

### What are the Perception Factors Creating Discrepancies in Risk Tolerance?

A separate factor analysis was conducted to determine the relationships within risk characteristic variables in different regions. The results (Table 7) were generally similar to the factor analysis results for all participants in hazard comparison analysis (Table 4).

**Table 7:** Factor Analysis Results of Risk Characteristics about HABs in Four Lakes.

Variables	Chaohu			Dianchi		
	Total VarianceExplained = 75%			Total VarianceExplained = 82%		
	Factor1*Knowledge*	Factor2*Effect*	Factor3*Trust*	Factor1*Knowledge*	Factor2*Effect*	Factor3*Trust*
Newness	**0.690**	0.065	−0.097	**0.801**	0.190	0.198
Immediacy	0.193	**0.621**	−0.103	0.104	**0.762**	−0.175
Social risk	−0.001	**0.794**	0.017	0.332	**0.693**	0.077
Personal effect	0.018	**0.753**	0.101	0.091	**0.583**	0.221
Knowledge	**0.671**	0.078	0.043	**0.604**	0.350	0.121
Benefit	−0.029	0.038	**0.980**	0.372	−0.009	**0.612**
Dread	0.292	**0.615**	0.142	0.084	**0.694**	0.139
Trust	0.072	−0.022	**0.712**	−0.096	0.043	**0.849**
**Variables**	**Hongze**			**Taihu**		
	**Total Variance** **Explained = 76%**			**Total Variance** **Explained = 79%**		
	**Factor1** ***Knowledge***	**Factor2** ***Effect***	**Factor3** ***Trust***	**Factor1** ***Knowledge***	**Factor2** ***Effect***	**Factor3** ***Trust***
Newness	**0.778**	0.099	−0.018	**0.789**	−0.048	−0.150
Immediacy	0.090	**0.654**	−0.121	0.308	**0.626**	0.100
Social risk	0.217	**0.707**	−0.058	−0.115	**0.724**	0.057
Personal effect	0.007	**0.625**	0.251	−0.117	**0.819**	0.026
Knowledge	**0.598**	0.314	0.001	**0.640**	0.058	−0.113
Benefit	0.337	0.072	**0.693**	−0.097	−0.038	**0.877**
Dread	−0.129	**0.725**	0.135	0.155	**0.775**	−0.046
Trust	0.228	−0.109	**0.763**	0.501	0.028	**0.589**

Note: See Table 3 for variables description. |Factor pattern|>0.40 is in boldface type.

Risk perception models were established to obtain the regression coefficients for respondents’ risk tolerance levels of HABs, using the three risk perception factors listed in Table 7 as independent variables (see Table 8). The results from the respondents around Lake Hongze and Lake Dianchi had weaker (i.e., less negative) correlations between *Effect* (Factor 2) and risk tolerance of HABs. *Knowledge* about hazards (Factor 1) and *Trust* in government or organizations (Factor 3) had positive coefficients, which indicated that tolerable levels of HABs increased as *Knowledge* and *Trust* increased, except in the Taihu area where there existed a significant negative association between *Knowledge* and risk tolerance. As a result, the impacts of these three factors on risk tolerance were not always of the same directions, especially the impacts of *Knowledge* (factor 1) and *Effect* (Factor 2). Overall, four perception models can explain 38.0%–52.2% of the variance in tolerable levels, and the independent variables considered in the risk perception model all played an important role in the decision process.

**Table 8:** Using Factor Scores about HABs to Explain Mean Risk Tolerance of Four Lakes.

Factors	Chaohu		Dianchi			Hongze			Taihu			
	*β*	SE	*β*	SE		*β*	SE		*β*		SE	
Factor1:*Knowledge*	0.226*	0.105	0.248**	0.040	0.245*		0.115	−**0.156** *		0.057		
Factor2:*Effect*	−**0.224** *	0.097	−0.061	0.019	−0.013		0.012	−**0.187** *		0.082		
Factor3:*Trust*	0.234*	0.113	0.348***	0.056	0.264**		0.098	0.237*		0.114		
*Adjusted R^2^*	0.485		0.522		0.380			0.401				
*F-value*	25.94***		27.86***		15.32***			23.51***				

* p<0.05,

** p<0.01,

*** p<0.001.

Note: The dependent variables are mean tolerance judgment for the HABs, calculated by averaging judgments over participants from different lakes. Unstandardized regression coefficients are from regression models with factor scores from the factor analysis as independent variables.

### How do Demographic Characteristics and Social Indicators Influence the Relationship between each Perception Factor and Risk Tolerance?

#### Impact of demographic variables on individual risk perception of HABs

**Figure 2:**
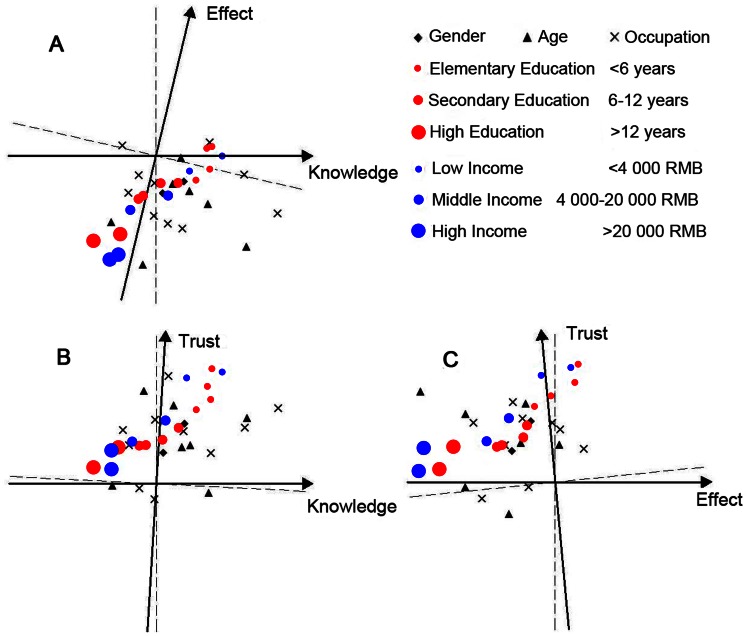
Individual risk perception factors for different demographic characteristics plotted in the factor space.

Table S1 shows the results of the regional demographics of the researched areas derived from the Kruskal-Wallis H (x^2^) test. To visualize the discrepancy in risk perception among different demographic characteristics, factors representing individual judgments of risk perception were plotted using factor space analysis. Fig. 2 reveals that more knowledge about a hazard is significantly associated with a higher risk tolerance level in low-income groups. However, it is worth noting that these correlations are significantly negative in high-income groups, indicating that among wealthy people, risk tolerance levels decrease as they learn more about a hazard. However, the factor *Knowledge* is not necessarily accurate: it only measures respondents’ perceptions of their levels of understanding of risks. Respondents may misgauge their own knowledge levels due to lack of access to relevant information.

**Figure 3:**
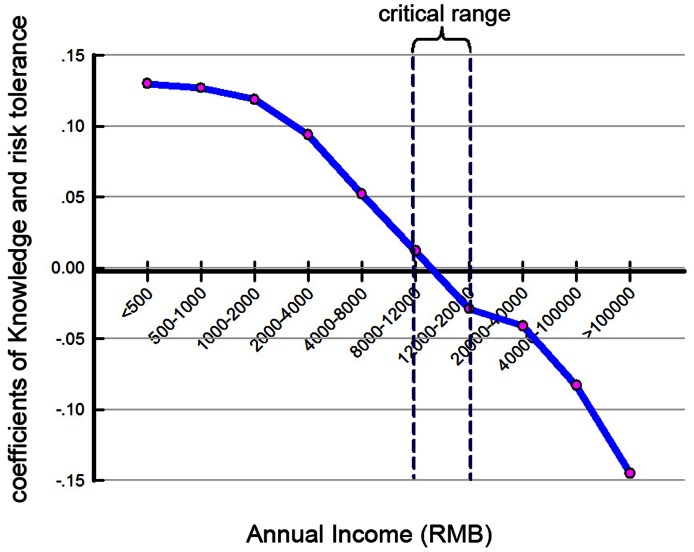
Regression analysis between annual income and the coefficients of Knowledge and risk tolerance.

Regression analysis was used to test the impact of an individual income on risk perception. The coefficients of *Knowledge* and risk tolerance- defined as the dependent variables- were categorized by annual income level, whose range was defined as the independent variable. The analysis revealed that annual income has a positive or negative correlation between *Knowledge* and risk tolerance within a critical range (8,000–20,000 RMB, or $1,250–$3,125 in U.S. dollars) (see Fig. 3).

#### Impact of social variables on public risk perception of HABs

**Figure 4:**
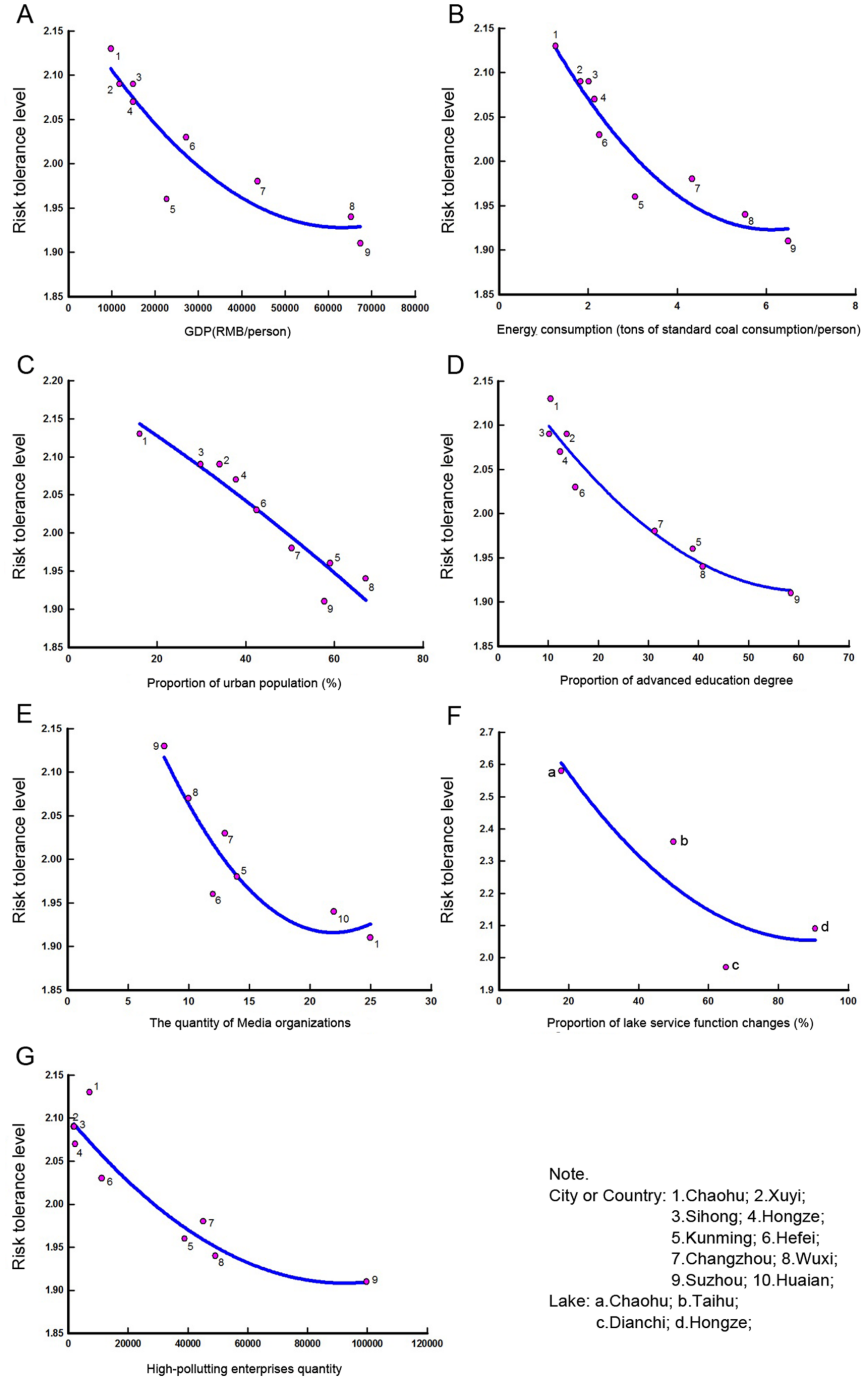
Regression analysis between social indicators and risk tolerance levels of HABs.

As the results shown in Fig. 4, significant relationships are existed between risk tolerance level of HABs and some social variables, especially energy consumption per capita (R^2^ = 0.91), and proportion of urban population (R^2^ = 0.91), and proportion of advanced education degrees (R^2^ = 0.94), which indicate that different socio-economic status or socio-educational level may correlated with different risk tolerance levels of local residents.

**Figure 5:**
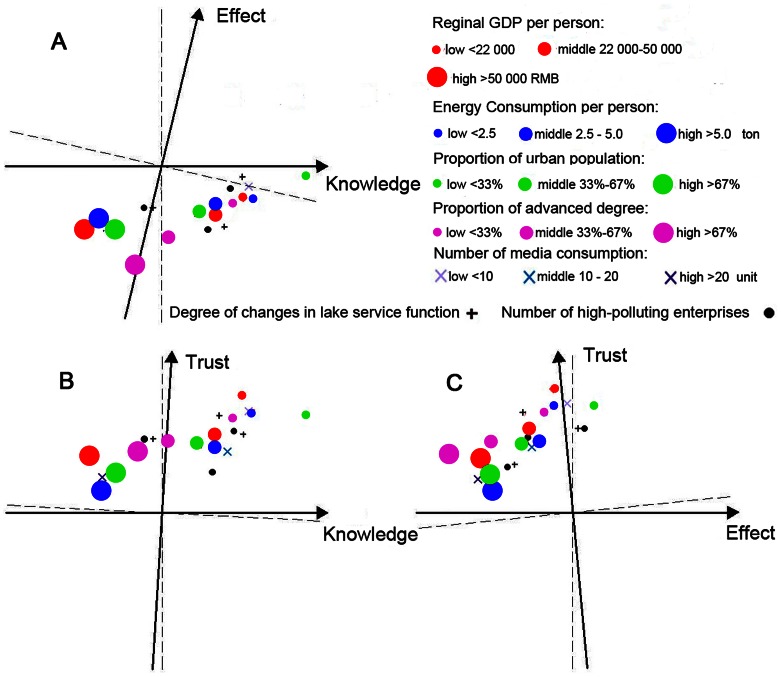
Public risk tolerance for different social conditions plotted in the factor space.

To visualize the diversity of risk perception in different social conditions, three risk perception factors were plotted with social variables in the factor space (Fig. 5). Clearly, in the regions with a low urban population or low GDP per capita, more Knowledge about the hazard was positively associated with higher risk tolerance levels, which is consistent with the previous analysis of individual income levels; the factor *Effect* and risk tolerance levels showed no significant correlations. In the regions with a high proportion of advanced education degrees, we found that the factor *Effect* was significantly correlated with higher risk tolerance levels, which is consistent with the previous analysis of individuals’ education levels. However, the factor Trust had a more significant impact on risk tolerance levels in the regions with few media organizations, while the factor *Effect* had a more significant impact on tolerable risk levels in the regions with many media organizations. More information about regional variance of risk tolerance according to different perception factors can be found in Fig. 5.

## Discussion

Currently, public risk tolerance levels of HABs in the four researched areas were all comparatively low (M = 2.28, SD = 1.22). On one hand, risk eliminating measures should be strengthened to reach publicly tolerable levels; on the other hand, the government should help the public to make rational judgments regarding perceived risks, which could help prevent public unease and ensure their support for ecological protection policies. Thus, understanding the factors that determine risk tolerance is very important for protecting both the environment and public safety. This study of risk perception models applied to HABs found that Chinese residents’ risk tolerance levels depend primarily on three factors: *Knowledge* (i.e., how much does one know about this kind of risk?), *Effect* (i.e., how would this kind of risk affect humans or society?), and *Trust* (i.e., how much can residents trust the government or organizations?).

Furthermore, the impact of these three perception factors varied among the respondents from the four large lakes. We found that regional economic development could explain most of this variance. For example, a negative relationship existed between *Knowledge* and risk tolerance for the respondents from Lake Taihu, while the correlation was significant but in a positive relationship for respondents from the other three lakes. In the demographic analysis, the proportion of high-income groups in the region of Taihu (53%) is much higher than that in the other three regions (Chaohu, 33%; Dianchi, 24%; Hongze, 22%) (See Table S1). This explains why the regression coefficient for respondents from Lake Taihu is opposite to that of respondents from the other lakes. This phenomenon indicates that people whose annual income is lower than a critical range of 8,000–20,000 RMB ($1,250–$3,125 U.S. dollars) care more about their own livelihoods and less about the ecological system, and even ignored a familiar risk in HABs. Although they understand the HABs phenomenon, they do not feel it intolerable. But public attitudes can be changed with an improved economic status. Three socio-economic indicators revealed this tendency. The coefficients of the social variables were all negative (see Fig. 4-A, B, C), indicating that risk tolerance levels decreased as socio-economic levels increased. These results support the findings from previous studies by Bi et al. and Lind [46], [47], and can explain regional differences in public sensitivity to ecological changes in the pilot study (Table 2).

Educational level also had a significant impact on the sensitivity of risk perception, especially for *Effect*. It was found that *Effect* and risk tolerance levels had significant negative correlations in the groups of high and middle education levels while correlations in low education level groups were close to zero or insignificant (Fig. 2). In demographic analyses, the proportions of groups with higher education in the regions of Taihu (47%) and Chaohu (42%) were higher than those of Dianchi (29%) and Hongze (26%), which could explain the higher coefficients of the factor *Effect* in the perception models of the former two regions. At the same time, the impacts of socio-educational conditions, namely the proportion of advanced education degrees and quantity of media consumption, displayed similar trends (Fig. 5).

Economic development in the Lake Taihu area has reached the level of moderately developed countries, with an average annual income of about 20,000 RMB ($3,125 U.S. dollars) per person [38], which was above the critical range of annual income. In addition, the proportion of urban population and GDP per capita was at a relatively higher level. This confirmed that public risk perception and socio-economic factors hold a strong correlation. The results showed that the residents from Lake Taihu considered their living environment a high priority and paid more attention to health risks, which prompted them to have the lowest risk tolerance levels for HABs. Furthermore, higher socio-educational levels could promote more concerns about the consequences of ecological risks. After a theoretical disaster turned into a reality, risk tolerance levels sharply decreased, which may eventually lead to social disorder or public panic. Therefore, more investment in risk prevention measures and stronger policies than those contained in the government’s existing restoration plan of Lake Taihu are needed.

With lower levels of economic development in the Hongze and Dianchi areas yielding an average annual income of about 8,000 RMB ($1,250 U.S. dollars) per person [37], [41], the top priority of the public in those areas is rapid economic development, and more economic burdens from lake restoration investments could lead to public discontent. Respondents from these areas weakly considered the *Effect* of HABs and were insensitive to the negative consequences of environment problems and ecological risks. Their perceived risk tolerance was evidently correlated with *Trust* in government or organizations. As in previous studies about nuclear power risk perception in Japan [48] and China [49], insensitive people who had higher trust in government would judge the risk depending on heuristic processing [50], [51] which is generally irrational. So if the government lost its credibility, these groups of people, who tend not to rely on rational judgment, would sharply decrease their risk tolerance levels and may unnecessarily become alarmed. Sensitivity to risk will affect individual behavior [35], [52]. For example, Cabrera et al. [53] found that the sensitivity to health risk of pesticide will affect the farm workers’ behavior. In many exposure assessment studies [54], [55], researchers have found that individuals’ behaviors would result in the difference of actual risk. Huang et al. [56] and Marchwinska et al. [57] believed improving access to education and better risk communication to increase sensitivity will reduce high-risk behaviors and strengthen local governments’ credibility for risk reduction, control and management. So, the findings of this study suggested that existing political structures played an important role in risk tolerance [6]. In order to strengthen local governments’ credibility for risk control and management, it’s necessary to improve access to education and better risk communication for those biased people, offering accurate information and guiding them on adjusting behaviors to avoid high risks. According to the stepwise development of economy and education, gradual efforts should be made to eliminate ecological problems such as HABs in lakes.

These results provided some insight into how policymakers can target certain groups for effective risk communication and public education programs. For example, public communicators may effectively achieve better aims by targeting different groups of people divided by income levels. Thus, risk communication can not only prevent inattentive or unwitting individuals from ignoring the genuine level of risk, but it can also raise public awareness of environmental protection and attract more attention to ecological changes, thereby reducing individual activities (such as tourism, farming, etc.) that degrade the ecological environment. In addition, the results may also suggest possible social discontent occurring in different sites according to the relationships between risk tolerance and social characteristics.

This study explores Chinese public perception of a specific eutrophication problem occurring in lake ecosystems, a phenomenon which has rarely concerned researchers but is gradually becoming one of the most serious ecological risks in China. The importance of this study lies in the finding that Chinese residents hailing from different regions may have different cognitions and perceptions about ecological risks, which suggests that regional characteristics influence public attitudes towards ecological problems. Due to complex social conditions and the sensitive conformist nature of the Chinese public, it is crucial to detect target groups in different regions, which helps to promote effective risk communication in improving ecosystem health and maintaining social stability.

Nevertheless, some important factors related to the risk perception of HABs still need to be addressed with further research, including emotion, action tendency, and willingness-to-pay. The role of many social factors and individual demographic variables also has not been sufficiently studied. Information about the participant’s factual knowledge of HAB risk which will be used to examine the factual correctness of their knowledge has not been collected. In addition, the Chinese public’s perception of different kinds of hazards may change significantly over time. All of these issues should be considered and discussed in future studies.

## Supporting Information

Table S1 Demographic Data of Study Participants in the Four Lakes.
(DOCX)

Table S2 Proportion of lake service function changes.
(DOCX)

Table S3 Limit for Environmental Quality Standard for Surface Water (mg/L).
(DOCX)

Questionnaire S1 (DOCX)
