# Supplementary material for: How Do the Chinese Perceive Ecological Risk in Freshwater Lakes?
Source: PLoS One. 2013 May 9;8(5):e62486. doi: 10.1371/journal.pone.0062486 (PMC3650014; doi:10.1371/journal.pone.0062486)
Supplement: Table S1 — Demographic Data of Study Participants in the Four Lakes. (DOCX) [file pone.0062486.s001.docx]

Table S1 Demographic Data of Study Participants in the Four Lakes

|  | | Chaohu | | Dianchi | | Hongze | | Taihu | | *X*^2^ test (p) |
| --- | --- | --- | --- | --- | --- | --- | --- | --- | --- | --- |
|  |  | Percent (%) | | Percent (%) | | Percent (%) | | Percent (%) | |  |
|  |  | N=339 ^a^ | Local population^b^ | N=267 ^a^ | Local population^b^ | N=315^a^ | Local population^b^ | N=440^a^ | Local population^b^ |  |
| Gender | Male | 44 | 50 | 41 | 50 | 53 | 52 | 52 | 51 | 0.012 (0.889) |
|  | Female | 56 | 50 | 59 | 50 | 47 | 48 | 48 | 49 |  |
| Age (years) | 16-19 | 9 | 15 | 5 | 10 | 9 | 12 | 12 | 10 | 4.269 (0.482) |
|  | 20-29 | 38 | 80 | 36 | 78 | 31 | 83 | 32 | 86 |  |
|  | 30-39 | 31 |  | 16 |  | 35 |  | 36 |  |  |
|  | 40-49 | 15 |  | 15 |  | 16 |  | 14 |  |  |
|  | 50-59 | 6 |  | 14 |  | 7 |  | 5 |  |  |
|  | >60 | 2 | 5 | 14 | 12 | 2 | 5 | 1 | 4 |  |
| Education (years) | ≤3 | 4 | 7 | 2 | 5 | 2 | 9 | 2 | 2 | **11.518^**^ (0.008)** |
|  | 3-6 | 4 | 26 | 8 | 34 | 15 | 31 | 3 | 27 |  |
|  | 6-9 | 22 | 36 | 31 | 35 | 25 | 39 | 23 | 40 |  |
|  | 9-12 | 28 | 18 | 30 | 15 | 33 | 14 | 25 | 18 |  |
|  | 12-16 | 38 | 13 | 26 | 11 | 24 | 7 | 40 | 13 |  |
|  | >16 | 4 |  | 3 |  | 1 |  | 7 |  |  |
| Occupation | Officer | 5 | 3 | 8 | - | 11 | 7 | 1 | 3 | 8.524 (0.162) |
|  | Enterprise employee | 14 | 10 | 15 | - | 25 | 19 | 21 | 16 |  |
|  | Self-employed | 22 | 83 | 13 | - | 27 | 16 | 24 | 26 |  |
|  | Farmer | 3 |  | 3 | - | 6 | 10 | 9 | 13 |  |
|  | Fisher folk | 12 |  | 4 | - | 4 | 43 | 6 | 10 |  |
|  | Unemployed | 5 |  | 5 | - | 5 |  | 3 | 2 |  |
|  | The retired | 5 |  | 21 | - | 2 |  | 5 | 6 |  |
|  | Housewife | 3 |  | 6 | - | 3 |  | 6 | 8 |  |
|  | Student | 25 |  | 24 | - | 12 |  | 13 | 10 |  |
|  | Other | 6 | 4 | 1 | - | 5 | 5 | 3 | 6 |  |
| Annual Income (RMB) | ≤500 | 2 | 60 | 5 | 60 | 5 | 60 | 1 | 60 | **29.963^***^ (0.000)** |
|  | 501-1,000 | 7 |  | 8 |  | 9 |  | 4 |  |  |
|  | 1,001-2,000 | 7 |  | 14 |  | 14 |  | 5 |  |  |
|  | 2,001-4,000 | 10 |  | 13 |  | 6 |  | 6 |  |  |
|  | 4,001-8,000 | 11 |  | 12 |  | 12 |  | 5 |  |  |
|  | 8,001-12,000 | 12 |  | 14 |  | 13 |  | 10 |  |  |
|  | 12,001-20,000 | 18 |  | 10 | 40 | 19 |  | 16 |  |  |
|  | 20,001-40,000 | 21 | 40 | 21 |  | 16 | 40 | 27 |  |  |
|  | 40,001-100,000 | 9 |  | 2 |  | 5 |  | 23 | 40 |  |
|  | >100,000 | 3 |  | 1 |  | 1 |  | 3 |  |  |

^a^: Fully completed questionnaires.

^b^: Data of local population

**p<0.01, ***p<0.001.
