# Supplementary material for: How Do the Chinese Perceive Ecological Risk in Freshwater Lakes?
Source: PLoS One. 2013 May 9;8(5):e62486. doi: 10.1371/journal.pone.0062486 (PMC3650014; doi:10.1371/journal.pone.0062486)
Supplement: Table S2 — Proportion of lake service function changes. (DOCX) [file pone.0062486.s002.docx]

**Table S2 Proportion of lake service function changes**

|  | Chaohu | Dianchi | Hongze | Taihu |
| --- | --- | --- | --- | --- |
| Changes (%) | 17.5 | 65.5 | 90.9 | 50.0 |
| $Changes=S_{loss}/\left( V_{total}+S_{loss} \right)$;  $V_{total}=V_{p}+V_{e}+V_{s}+V_{c}$;  $S_{loss}=S_{p}+S_{e}+S_{s}+S_{c}$;  *V_p_*: the value of material production that lake ecosystem provide;  *V_e_*: the value of environmental adjustment that lake ecosystem provide;  *V_s_*: the value of life supporting that lake ecosystem provide;  *V_c_*: the value of culture services that lake ecosystem provide;  *S_p_*: the loss in value of material production that lake ecosystem provide;  *S_e_*: the loss in value of environmental adjustment that lake ecosystem provide;  *S_s_*: the loss in value of life supporting that lake ecosystem provide;  *S_c_*: the loss in value of culture services that lake ecosystem provide; | | | | |

Note: The concrete data are not publicly released for compliance with the report requirements.
