# Supplementary material for: How Do the Chinese Perceive Ecological Risk in Freshwater Lakes?
Source: PLoS One. 2013 May 9;8(5):e62486. doi: 10.1371/journal.pone.0062486 (PMC3650014; doi:10.1371/journal.pone.0062486)
Supplement: Table S3 — Limit for Environmental Quality Standard for Surface Water (mg/L). (DOCX) [file pone.0062486.s003.docx]

**Table S3 Limit for Environmental Quality Standard for Surface Water (mg/L)**

| No. | Class | | Ⅰ | Ⅱ | Ⅲ | Ⅳ | Ⅴ |
| --- | --- | --- | --- | --- | --- | --- | --- |
|  | Standard | |  |  |  |  |  |
|  | Item | |  |  |  |  |  |
| 1 | Water Temperature (℃) |  | Man-made environmental temperature change should be limited in:  Maximum weekly average temperature rise ≤ 1  Maximum weekly average temperature drop ≤ 2 | | | | |
| 2 | pH |  | 6-9 | | | | |
| 3 | Dissolved Oxygen | ≥ | saturation ratio 90%  (or 7.5) | 6 | 5 | 3 | 2 |
| 4 | Permanganate Index | ≤ | 2 | 4 | 6 | 10 | 15 |
| 5 | Chemical Oxygen Demand (COD) | ≤ | 15 | 15 | 20 | 30 | 40 |
| 6 | BOD_5_ | ≤ | 3 | 3 | 4 | 6 | 10 |
| 7 | NH_3_-N | ≤ | 0.15 | 0.5 | 1.0 | 1.5 | 2.0 |
| 8 | Total Phosphorus（as P） | ≤ | 0.02  (lake/reservoir 0.01) | 0.1  (lake/reservoir 0.025) | 0.2  (lake/reservoir 0.05) | 0.3  (lake/reservoir 0.1) | 0.4  (lake/reservoir 0.2) |
| 9 | Total Nitrogen (as N in reservoir and lake) | ≤ | 0.2 | 0.5 | 1.0 | 1.5 | 2.0 |
| 10 | Cu | ≤ | 0.01 | 1.0 | 1.0 | 1.0 | 1.0 |
| 11 | Zn | ≤ | 0.05 | 1.0 | 1.0 | 2.0 | 2.0 |
| 12 | Fluoride (as F^-^) | ≤ | 1.0 | 1.0 | 1.0 | 1.5 | 1.5 |
| 13 | Se | ≤ | 0.01 | 0.01 | 0.01 | 0.02 | 0.02 |
| 14 | As | ≤ | 0.05 | 0.05 | 0.05 | 0.1 | 0.1 |
| 15 | Hg | ≤ | 0.00005 | 0.00005 | 0.0001 | 0.001 | 0.001 |
| 16 | Cd | ≤ | 0.001 | 0.005 | 0.005 | 0.005 | 0.01 |
| 17 | Cr (Ⅵ) | ≤ | 0.01 | 0.05 | 0.05 | 0.05 | 0.1 |
| 18 | Pb | ≤ | 0.01 | 0.01 | 0.05 | 0.05 | 0.1 |
| 19 | Cyanide | ≤ | 0.005 | 0.05 | 0. 2 | 0.2 | 0.2 |
| 20 | Volatile Phenol | ≤ | 0.002 | 0.002 | 0.005 | 0.01 | 0.1 |
| 21 | Petroleum | ≤ | 0.05 | 0.05 | 0.05 | 0.5 | 1.0 |
| 22 | An-ionic Surfactant | ≤ | 0.2 | 0.2 | 0.2 | 0.3 | 0.3 |
| 23 | Sulfide | ≤ | 0.05 | 0.1 | 0.2 | 0.5 | 1.0 |
| 24 | Fecal Coliform（/L） | ≤ | 200 | 2000 | 10000 | 20000 | 40000 |

Environmental Quality Standard for Surface Water (GB3838-2002)

The Standard is jointly issued by the State Environmental Protection Administration (SEPA) and the General Administration of Quality Supervision, Inspection and Quarantine of the People's Republic of China (AQSIQ).
