# Supplementary material for: How Do the Chinese Perceive Ecological Risk in Freshwater Lakes?
Source: PLoS One. 2013 May 9;8(5):e62486. doi: 10.1371/journal.pone.0062486 (PMC3650014; doi:10.1371/journal.pone.0062486)
Supplement: Questionnaire S1 — (DOCX) [file pone.0062486.s004.docx]

Questionnaire S1

The pictures below clearly showed that the water quality of lake a decade ago were quite clean and surrounded by the lush vegetation everywhere.


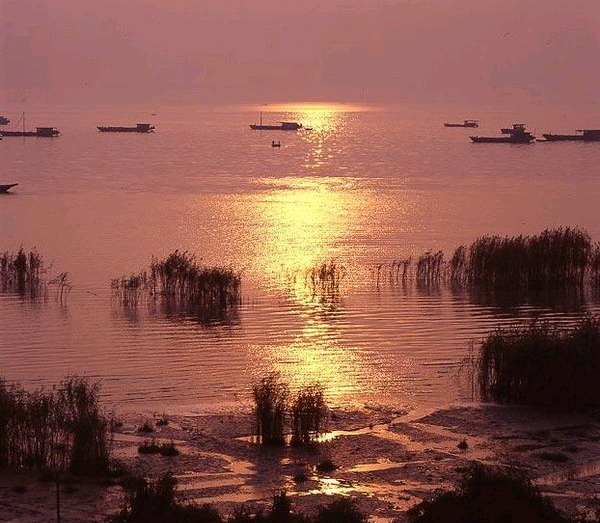


In recent years, the water quality deteriorated severely (water transparency decreased, algae bloom occurred frequently, plants of lake riparian decreased) resulting in serious consequences.


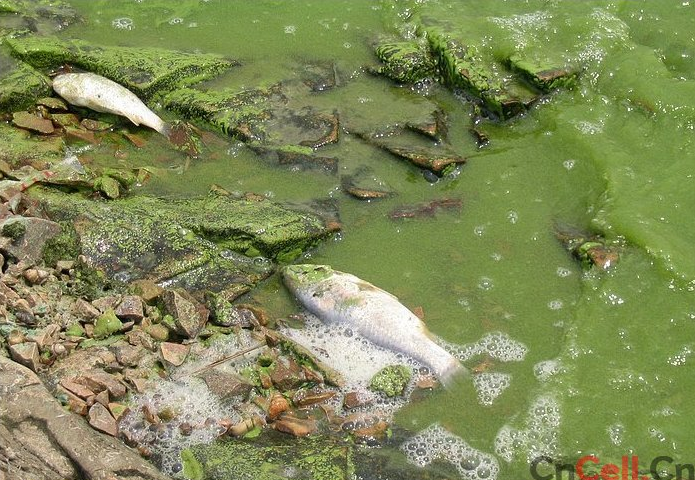

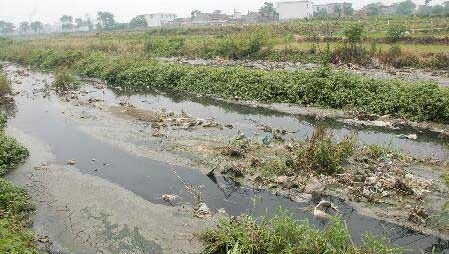


1. **Perceptions of ecological change**

1 Do you feel that the water quality of the lake has changed in the last decade?

1. Much improved ② Improved but not obvious ③ Unchanged ④ Deteriorated but not obvious ⑤ Much worsened

2 Have you noticed that the lake’s harmful algal blooms are occurring more frequently than a decade of ago?

1. Strongly disagree ② Disagree ③ Neutral ④ Agree ⑤ Strongly agree

3 Do you feel that the area of wetlands surrounding the lake has changed in the last decade?

1. much increased ② Increased but not obvious ③ Unchanged ④ Decreased but not obvious ⑤ much decreased

4 Do you feel that the number species of fish in the lake has been changed in the last decade?

1. much increased ② Increased but not obvious ③ Unchanged ④ Decreased but not obvious ⑤ much decreased

5 Do you feel that the number of species of birds inhabiting this lake has changed in the last decade?

1. much increased ② Increased but not obvious ③ Unchanged ④ Decreased but not obvious ⑤ much decreased
2. Do you feel that the number of species of water plants in the lake has changed in the last decade?

① much increased ② Increased but not obvious ③ Unchanged ④ Decreased but not obvious ⑤ much decreased

1. Do you feel that the number of tourists to this lake has changed in the last decade?

① much increased ② Increased but not obvious ③ Unchanged ④ Decreased but not obvious ⑤ much decreased

1. Do you feel that the number of inhabitants living around the lake has changed in the last decade?

① much increased ② Increased but not obvious ③ Unchanged ④ Decreased but not obvious ⑤ much decreased

1. **perceptions of risk characteristics**

Via the following questions, we will investigate residents’ perceived levels of environmental risks around them in order to remind them to pay more attention to their environment and face the remaining risks with right attitude. Then, we will make our lives healthier and the environment more beautiful.

(Please tick the option ① ② ③ ④ ⑤ that fits for you)

**Harmful algae bloom:**

The lake eutrophication tends to cause algae bloom and seriously threatens the function of water bodies. A 2007 algal bloom event in Taihu affected nearby citizens’ domestic water and may potentially cause various diseases. Meanwhile, the deterioration of the water quality decreased the quantity and quality of the aquatic products in lakes as well as destroyed the biodiversity. What’s more, HABs produce potent Cyanobacteria toxins that pose health threats to humans and aquatic organism, through ingestion or inhalation of the toxins. Cyanobacteria toxins may cause not only acute poisoning (nausea, diarrhea, lack of motor coordination, pupil dilation, etc.) but also cancer due to chronic exposure to low concentrations in drinking water.

| Questions | Value |
| --- | --- |
| 1. Is the risk associated with each activity, substance, or technology new and non-familiar, or is it old and familiar? | Old ①②③④⑤ New |
| 1. Are the effects of the risk associated with each activity, substance, or technology immediate, or will they take place in the future? | Occurs Immediately ①②③④⑤ Occurs far in the future |
| 1. How much risk is the national population subjected to as a product of each activity, substance, or technology? | No risk ①②③④⑤ High risk |
| 1. In what degree are you personally affected by the risk associated to each activity, substance, or technology? | Doesn’t affect me ①②③④⑤ Affects me |
| 1. To what degree is the risk associated with each activity, substance, or technology known to you? | No knowledge ①②③④⑤ High level of knowledge |
| 1. How beneficial to you is the use, consumption, or accomplishment of each activity, substance or technology? | Low ①②③④⑤ High |
| 1. Is the risk associated with each activity, substance, or technology a common risk or a terrible risk? | Common①②③④⑤ Terrible |
| 1. To what degree do you trust in the government or organizations? | No Trust at all ①②③④⑤ Complete trust |
| 1. If your life or working surroundings contain this kind of risk, what is your tolerable degree of risk? | Not tolerable at all ①②③④⑤ Very tolerable |

**Earthquakes:**

The earthquake is the earth surface vibration of fast and a kind of natural disasters. Consequences of the earthquakes vary depending on earthquake magnitudes, such as the 1976 7.8-magnitude earthquake in Tangshan, Hebei, China, 70% to 80% of the buildings collapsed and suffered heavy casualties. The recent earthquake in Wenchuan, Sichuan recorded 8.0-magnitude having caused serious casualties.

| Questions | Value |
| --- | --- |
| 1. Is the risk associated with each activity, substance, or technology new and non-familiar, or is it old and familiar? | Old ①②③④⑤ New |
| 1. Are the effects of the risk associated with each activity, substance, or technology immediate, or will they take place in the future? | Occurs Immediately ①②③④⑤ Occurs far in the future |
| 1. How much risk is the national population subjected to as a product of each activity, substance, or technology? | No risk ①②③④⑤ High risk |
| 1. In what degree are you personally affected by the risk associated to each activity, substance, or technology? | Doesn’t affect me ①②③④⑤ Affects me |
| 1. To what degree is the risk associated with each activity, substance, or technology known to you? | No knowledge ①②③④⑤ High level of knowledge |
| 1. How beneficial to you is the use, consumption, or accomplishment of each activity, substance or technology? | Low ①②③④⑤ High |
| 1. Is the risk associated with each activity, substance, or technology a common risk or a terrible risk? | Common①②③④⑤ Terrible |
| 1. To what degree do you trust in the government or organizations? | No Trust at all ①②③④⑤ Complete trust |
| 1. If your life or working surroundings contain this kind of risk, what is your tolerable degree of risk? | Not tolerable at all ①②③④⑤ Very tolerable |

**Nuclear power:**

Nuclear technology is used to produce electricity which can alleviate electric pressure, reduce prices and reduce greenhouse gas emissions. Since the first nuclear power plant’s running, two serious accidents happened because of nuclear power plants which affected the environment and human security. One is an nuclear accident at Three Mile Island in America in March 1979. Another is at Chernobyl in the Soviet Union in April 1986.

| Questions | Value |
| --- | --- |
| 1. Is the risk associated with each activity, substance, or technology new and non-familiar, or is it old and familiar? | Old ①②③④⑤ New |
| 1. Are the effects of the risk associated with each activity, substance, or technology immediate, or will they take place in the future? | Occurs Immediately ①②③④⑤ Occurs far in the future |
| 1. How much risk is the national population subjected to as a product of each activity, substance, or technology? | No risk ①②③④⑤ High risk |
| 1. In what degree are you personally affected by the risk associated to each activity, substance, or technology? | Doesn’t affect me ①②③④⑤ Affects me |
| 1. To what degree is the risk associated with each activity, substance, or technology known to you? | No knowledge ①②③④⑤ High level of knowledge |
| 1. How beneficial to you is the use, consumption, or accomplishment of each activity, substance or technology? | Low ①②③④⑤ High |
| 1. Is the risk associated with each activity, substance, or technology a common risk or a terrible risk? | Common①②③④⑤ Terrible |
| 1. To what degree do you trust in the government or organizations? | No Trust at all ①②③④⑤ Complete trust |
| 1. If your life or working surroundings contain this kind of risk, what is your tolerable degree of risk? | Not tolerable at all ①②③④⑤ Very tolerable |

**Public traffic:**

People need vehicles running on roads in their daily life which may cause casualties or property losses through errors or something untoward.

| Questions | Value |
| --- | --- |
| 1. Is the risk associated with each activity, substance, or technology new and non-familiar, or is it old and familiar? | Old ①②③④⑤ New |
| 1. Are the effects of the risk associated with each activity, substance, or technology immediate, or will they take place in the future? | Occurs Immediately ①②③④⑤ Occurs far in the future |
| 1. How much risk is the national population subjected to as a product of each activity, substance, or technology? | No risk ①②③④⑤ High risk |
| 1. In what degree are you personally affected by the risk associated to each activity, substance, or technology? | Doesn’t affect me ①②③④⑤ Affects me |
| 1. To what degree is the risk associated with each activity, substance, or technology known to you? | No knowledge ①②③④⑤ High level of knowledge |
| 1. How beneficial to you is the use, consumption, or accomplishment of each activity, substance or technology? | Low ①②③④⑤ High |
| 1. Is the risk associated with each activity, substance, or technology a common risk or a terrible risk? | Common①②③④⑤ Terrible |
| 1. To what degree do you trust in the government or organizations? | No Trust at all ①②③④⑤ Complete trust |
| 1. If your life or working surroundings contain this kind of risk, what is your tolerable degree of risk? | Not tolerable at all ①②③④⑤ Very tolerable |

1. **Demographic variables**

1 Gender: ①male ②female

2 Age: _________ years old

3 How many people are there in your family? ______

4 Your education level:

① never schooled ② primary school ③ middle school ④ high school/vocational technical institute ⑤ college/junior college ⑥ postgraduate

5 Your profession:

①Civil Servant ②Enterprise Employee ③the Self-employed ④Farmer ⑤Fisherman ⑥the Unemployed ⑦Retiree/Housewife⑧Student ⑨Others (details)_______

6 Your family’s income in 2007.

①less than 500 ②501-1 000 ③1 001-2 000 ④2 001- 4 000 ⑤4 001- 8 000 ⑥8 001-12 000 ⑦12 001- 20 000 ⑧20 001- 40 000 ⑨40 001- 100 000 ⑩more than 100 001

Thanks for your participation!
